# Potential role of maintaining physiological testosterone levels in improving glucose metabolism among normal-weight Japanese women: a pilot exploratory study

**DOI:** 10.3389/fendo.2026.1878776

**Published:** 2026-07-06

**Authors:** Takahiro Tsutsumi, Saki Komai, Asako Miyazaki, Kyoichiro Tsuchiya

**Affiliations:** 1 Department of Diabetes and Endocrinology, Graduate School of Interdisciplinary Research, Faculty of Medicine, University of Yamanashi, Chuo, Yamanashi, Japan; 2 Kua-house Isawa, Isawa Hot Spring Hospital, Fuefuki, Yamanashi, Japan

**Keywords:** glucose metabolism, insulin resistance, normal-weight, testosterone, women’s health

## Abstract

**Background:**

Traditionally, elevated androgen levels in women have been associated with obesity and deteriorated glucose metabolism. However, most existing data are derived from patients with polycystic ovary syndrome (PCOS) or obesity. Consequently, the physiological role of maintaining testosterone levels in normal-weight women remains poorly understood.

**Objective:**

To eliminate metabolic biases associated with obesity, this exploratory study investigated the relationship between serum total testosterone levels and parameters of body fat and glucose metabolism (HbA1c, fasting plasma glucose, and HOMA-IR) specifically in normal-weight Japanese women (BMI 18.5–25 kg/m²).

**Methods:**

Out of 100 Japanese women who underwent health checkups, 69 normal-weight individuals were included in the analysis. Correlations between total testosterone levels and glucose metabolism indices were examined.

**Results:**

Total testosterone levels showed significant inverse correlations with HbA1c (*ρ* = -0.341, *p* = 0.004), fasting glucose (*ρ* = -0.327, *p* = 0.006), and HOMA-IR (*ρ* = -0.274, *p* = 0.023). In multivariate regression analysis, these inverse associations remained significant for HbA1c and fasting glucose after adjusting for BMI and lifestyle factors. Conversely, while the associations with HbA1c and fasting glucose were attenuated after adjusting for age and menopausal status, the inverse association between total testosterone and HOMA-IR remained independently significant after adjusting for age (*β* = -0.178, *p* = 0.029) and further for menopausal status (*β* = -0.173, *p* = 0.032).

**Conclusion:**

This study demonstrates that even in normal-weight women, low total testosterone levels are associated with impaired glucose metabolism. These results suggest that, in the absence of obesity-induced androgen excess, the maintenance of physiological androgen levels may contribute to metabolic maintenance in women. These findings suggest a novel perspective on female metabolic health, highlighting the potential importance of physiological androgen homeostasis in the regulation of glucose metabolism.

## 1 Introduction

It is widely recognized that androgens, including testosterone, impair glucose metabolism in women, clinically manifesting as insulin resistance and polycystic ovary syndrome (PCOS) (1, 2). Unlike in men, where over 95% of testosterone is synthesized in the testes, a significant portion of female androgens is produced in peripheral tissues, such as adipose tissue; consequently, increased adiposity directly serves as a major source for androgen elevation (3, 4). Furthermore, obesity-induced insulin resistance acts as a potent stimulator of ovarian androgen production, further exacerbating circulating testosterone levels. This obesity-driven hyperandrogenemia promotes further adiposity and induces a vicious cycle of metabolic deterioration (5, 6). Consequently, the paradigm of “a pathological sequence involving obesity, androgen excess, and subsequent metabolic deterioration” has become dominant (1, 7). In this context of pathological hyperandrogenism, such as in patients with PCOS, extensive research is underway to elucidate distinct pathophysiological mechanisms using specific indices like the total testosterone to dihydrotestosterone (TT/DHT) ratio as biomarkers for metabolic impairment (8). Despite these ongoing advancements in understanding pathological states, evidence regarding the impact of maintaining physiological, non-excessive androgen levels on glucose metabolism in non-obese women remains deficient.

In men, it is well established that preserving physiological androgen levels is protective against metabolic dysfunction by suppressing excessive adiposity (9, 10). Furthermore, at the cellular level, androgens promote insulin secretion in the pancreas and maintain insulin sensitivity in skeletal muscle via androgen receptor (AR) signaling (11, 12). Therefore, similar to the divergent effects of thyroid hormones on glucose metabolism depending on whether levels are physiological or excessive (13), it is plausible that maintaining physiological levels of androgens in women may not necessarily be detrimental and could even play a role in supporting optimal glucose metabolism.

**Figure 1:**
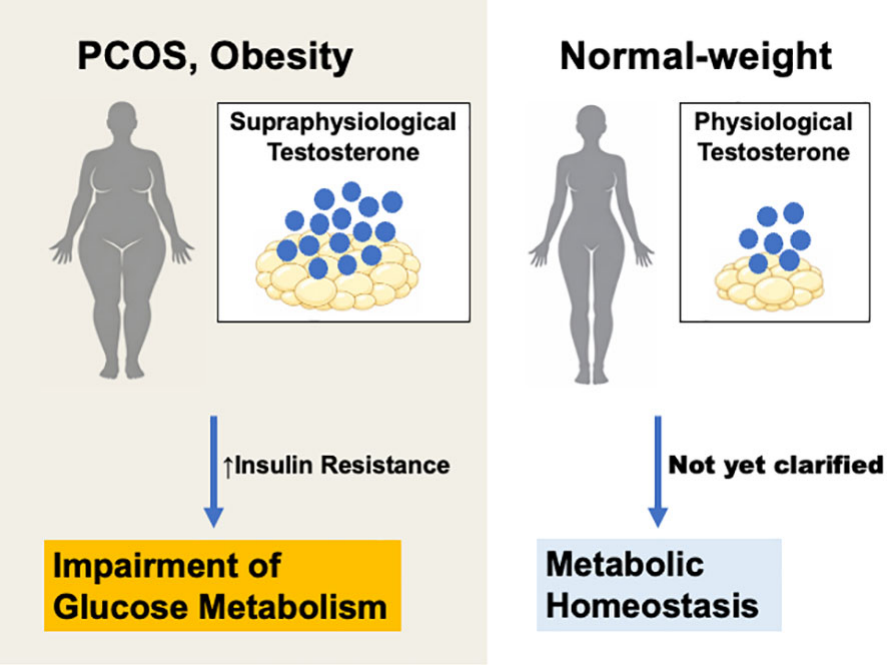
Proposed mechanisms linking testosterone status and glucose metabolism parameters across different body weights. The schematic illustrates the relationship between testosterone levels and metabolic status. Adipose tissue functions as a site for peripheral testosterone synthesis in women; therefore, obesity is associated with elevated testosterone levels. Yellow clusters represent adipocytes, and blue dots represent testosterone. Left (PCOS, Obesity): In women with PCOS and concurrent obesity, expanded adipose tissue and associated pathways lead to supraphysiological testosterone levels. This androgen excess contributes to heightened insulin resistance, subsequently resulting in the impairment of glucose metabolism. Right (Normal weight): In non-obese populations, such as normal-weight Japanese women, testosterone levels typically remain within the physiological range due to minimal adiposity. It remains not yet clarified whether maintaining testosterone homeostasis actively supports metabolic homeostasis independent of these confounding effects. PCOS, polycystic ovary syndrome.

To test this hypothesis, an analysis focusing on normal-weight women, who lack the confounding factors of obesity and obesity-induced insulin resistance that drive androgen elevation, is effective for exploring the potential metabolic significance of maintaining physiological androgen homeostasis. East Asians, including the Japanese population, have significantly lower obesity rates compared to Western populations, and unique mechanisms of glucose metabolism in non-obese individuals have been extensively reported in these populations (14). By focusing specifically on normal-weight Japanese women, this study aims to isolate metabolic impairment independent of obesity from androgen elevation associated with excess adiposity, thereby exploring the potential role of maintaining physiological androgen homeostasis in glucose metabolism (**Figure 1**).

This study was designed as an exploratory analysis with a deliberately focused sample size to ensure high data precision, population homogeneity, and the detection of robust effect sizes. We believe these findings serve as a critical foundation for validating this methodology and providing a rationale for future large-scale investigations.

## 2 Materials and methods

### 2.1 Study population

This cross-sectional study was conducted among 100 women who underwent elective health checkups between December 2023 and February 2024 at Kurhaus Isawa, a health screening facility in Yamanashi Prefecture, Japan. This facility provides standardized medical examinations for the general public. During the study period, all female examinees were initially considered potential candidates. Participants were excluded from the study if they met any of the following criteria: a diagnosis of hypopituitarism; current or recent (within the past six months) hormone replacement therapy, including sex steroid hormones; a diagnosis of congenital adrenal hyperplasia; or insufficient serum sample volume to perform the required biochemical analyses. Following the primary screening, medical staff provided comprehensive explanations of the study objectives and procedures to eligible candidates. Written informed consent was obtained from all participants who voluntarily agreed to participate prior to enrollment. The study protocol was approved by the Ethics Committee of the University of Yamanashi (Approval No. CS0064).

### 2.2 Data collection

Participant demographic and clinical characteristics—including age, body mass index (BMI), blood pressure, comorbidities, lifestyle factors, and menopausal status—were collected using health screening records and medical interview records. Standing height and body weight were measured to the nearest 0.1 cm and 0.1 kg, respectively, with participants wearing light clothing and no shoes. Height, body weight, and body fat were assessed using an automated bioelectrical impedance–based body composition analyzer (DC-270-N; TANITA Corp., Tokyo, Japan). BMI was calculated as body weight (kg) divided by height squared (m²).

Blood pressure was measured twice using an automated electronic sphygmomanometer (Elemano 2; Terumo Corp., Tokyo, Japan) after participants had rested in a seated position for at least 5 minutes, and the mean value was used for analysis.

Venous blood samples were collected between 7:00 and 10:00 a.m. after an overnight fast. Fasting plasma glucose, triglycerides, and lipoprotein cholesterol concentrations were measured using an automated biochemical analyzer (BioMajesty JCA-BM6050; JEOL Ltd., Tokyo, Japan). Hemoglobin A1c (HbA1c) levels were determined by high-performance liquid chromatography using an Adams Hybrid AH-8290 analyzer (ARKRAY Inc., Kyoto, Japan). Serum samples were aliquoted and stored at -80 °C until hormonal analyses were performed.

Serum total testosterone concentrations were measured by electrochemiluminescence immunoassay (ECLIA) at a certified commercial laboratory (SRL Inc., Tokyo, Japan). The assay had a limit of detection (LOD) of 0.03 ng/mL, and the intra- and inter-assay coefficients of variation were both <10%. Serum insulin concentrations were measured using a commercially available enzyme-linked immunosorbent assay (ELISA) kit (Mercodia AB, Uppsala, Sweden; catalog no. 10-1113-01) in accordance with the manufacturer’s instructions.

### 2.3 Sample size and statistical analysis

Based on the National Health and Nutrition Survey in Japan, the proportion of normal-weight Japanese women (BMI ≥ 18.5 and < 25.0 kg/m²) is estimated to be approximately 70% in adult women. To evaluate the primary endpoint—the correlation between total testosterone levels and glucose metabolism parameters (e.g., HOMA-IR)—the sample size was calculated assuming a significance level of 0.05 and a statistical power of 80%. Based on a previously reported correlation coefficient (Spearman’s *ρ* = 0.4) between hormonal dynamics and metabolic indices in non-obese women, the required sample size was estimated to be 47 participants (15). To ensure precision in specimen collection and to minimize variability attributable to seasonal fluctuations or differences in collection methods, the final sample size was determined as the maximum number of participants that could be enrolled at a single institution within a single season (16).

Data normality was assessed using the Shapiro–Wilk test. Continuous variables are presented as mean ± standard deviation for normally distributed data, or as median [interquartile range] for non-normally distributed data. To evaluate the association between total testosterone levels and glucose metabolism parameters, Spearman’s rank correlation analysis was initially conducted. Subsequently, multiple linear regression analysis was performed to assess the independent effects of total testosterone on glucose metabolism, adjusting for age, BMI, body fat, menopausal status, and lifestyle factors, including smoking status (0: never, 1: former, and 2: current), alcohol intake frequency (0, none; 1, 1 day/week; 2, 2–3 days/week; 3, 4–5 days/week; and 4, almost daily), and regular exercise habit (0: no, 1: yes). Multicollinearity was assessed using variance inflation factors (VIFs), and all VIF values were confirmed to be less than 5, indicating no significant multicollinearity among the independent variables. Variables with non-normal distributions, including total testosterone, HOMA-IR, BMI, and age, were log-transformed prior to inclusion in multivariate analyses. For total testosterone values below the limit of detection (LOD, 0.03 ng/mL), a simple substitution method using LOD/√2 was applied in the primary analysis (17). All statistical analyses were performed using Stata version 17.0 (StataCorp, College Station, TX, USA) and GraphPad Prism version 11.0.0 (GraphPad Software, San Diego, CA, USA). Statistical significance was defined as p < 0.05.

## 3 Results

### 3.1 Participant characteristics

Of the 100 original study participants, 69 women with a normal BMI were included in the final analysis (**Table 1**). The clinical characteristics of the study participants are as follows. The median age was 53.0 [45.0–66.0] years, and 38 participants (55.1%) were postmenopausal. The prevalence of preexisting diabetes mellitus was low at 2.9% (n = 2) (**Table 1**). Notably, the participants exhibited a lean profile with remarkably low insulin resistance, featuring a median BMI of 20.8 [19.8–22.5] kg/m², a mean body fat of 29.2 ± 4.1%, and a median HOMA-IR as low as 0.6 [0.4–0.9]. None of the participants reported a medical history of PCOS.

**Table 1:** Clinical characteristics of normal-weight women.

Characteristics	*n* = 69
Age (years)	53.0 [45.0–66.0]
Height (cm)	156.9 ± 5.3
Weight (kg)	52.1 ± 5.0
BMI (kg/m^2^)	20.8 [19.8–22.5]
Body fat (%)	29.2 ± 4.1
Systolic blood pressure (mmHg)	113.4 ± 14.0
Diastolic blood pressure (mmHg)	72.5 ± 9.1
HbA1c (%)	5.6 ± 0.3
Fasting glucose (mg/dL)	94.5 ± 7.3
Fasting insulin (μIU/mL)	2.6 [1.7–4.0]
HOMA-IR	0.6 [0.4–0.9]
Triglycerides (mg/dL)	70.0 [50.5–91.5]
Total cholesterol (mg/dL)	201.0 [183.0–226.5]
HDL-cholesterol (mg/dL)	71.5 ± 13.9
LDL-cholesterol (mg/dL)	127.8 ± 28.4
Total testosterone (ng/mL)	0.14 [0.07–0.22]
Menopausal status, *n* (%)	38 (55.1)
Diabetes, *n* (%)	2 (2.9)

Values are presented as mean ± SD or median (25th–75th percentiles).

BMI, body mass index; HbA1c, hemoglobin A1c; HDL-cholesterol, high-density lipoprotein cholesterol; HOMA-IR, homeostasis model assessment of insulin resistance; LDL-cholesterol, low-density lipoprotein cholesterol.

### 3.2 Correlation between total testosterone and markers of glucose metabolism

**Figure 2:**
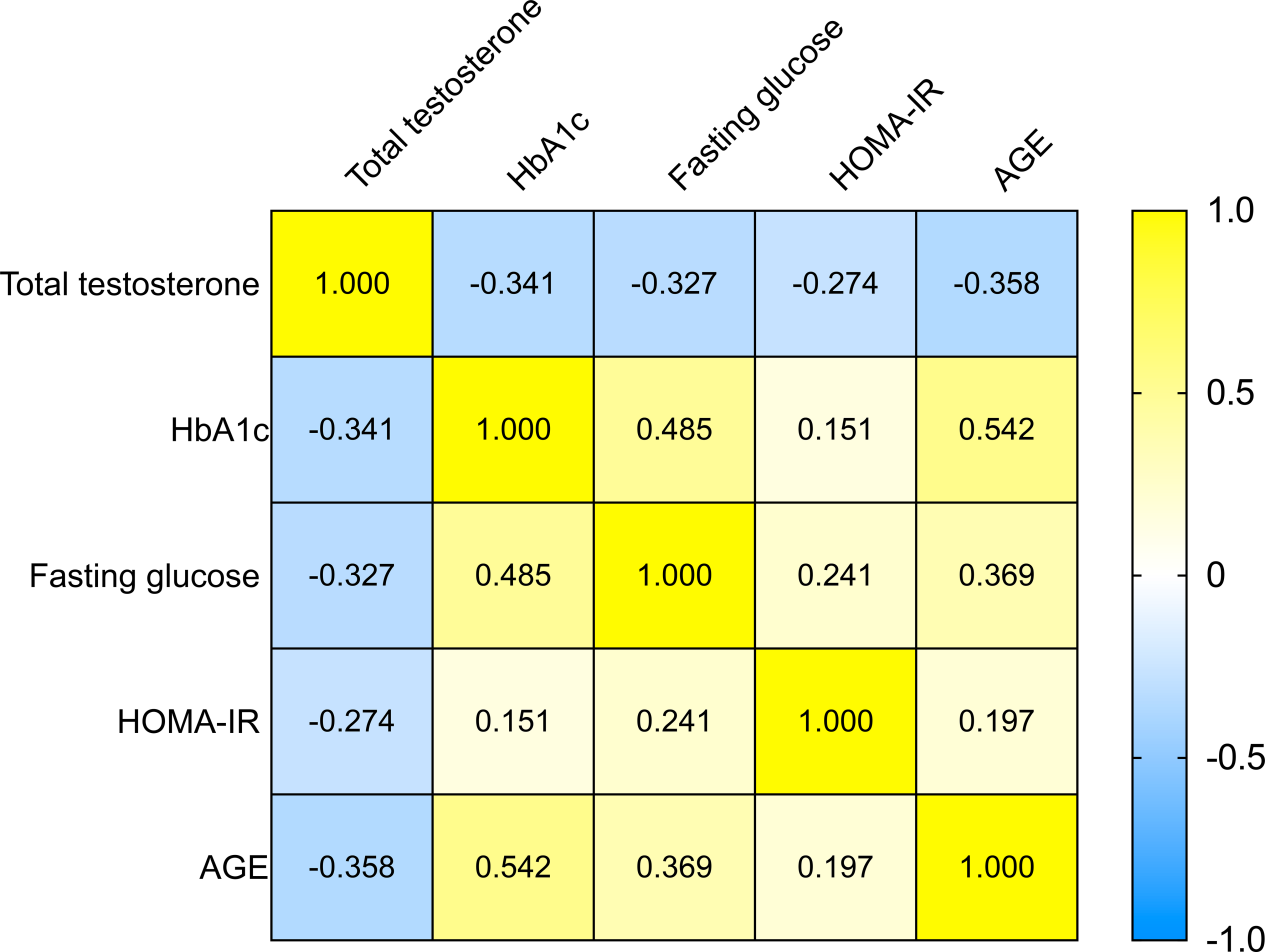
Correlation matrix between total testosterone, glucose metabolism parameters, and age. The heatmap displays Spearman’s rank correlation coefficients (*ρ*) between variables. The statistical analysis included the entire cohort (n = 69), with 13 subjects whose total testosterone levels were below the limit of detection (LOD) treated as the lowest rank. The color scale on the right indicates the strength of the correlation, with yellow representing a positive correlation and blue representing a negative (inverse) correlation. Total testosterone levels showed significant inverse correlations with all glucose metabolism parameters (HbA1c, fasting glucose, and HOMA-IR) as well as age (all *p* < 0.05). HbA1c, hemoglobin A1c; HOMA-IR, homeostasis model assessment of insulin resistance.

**Figure 3:**
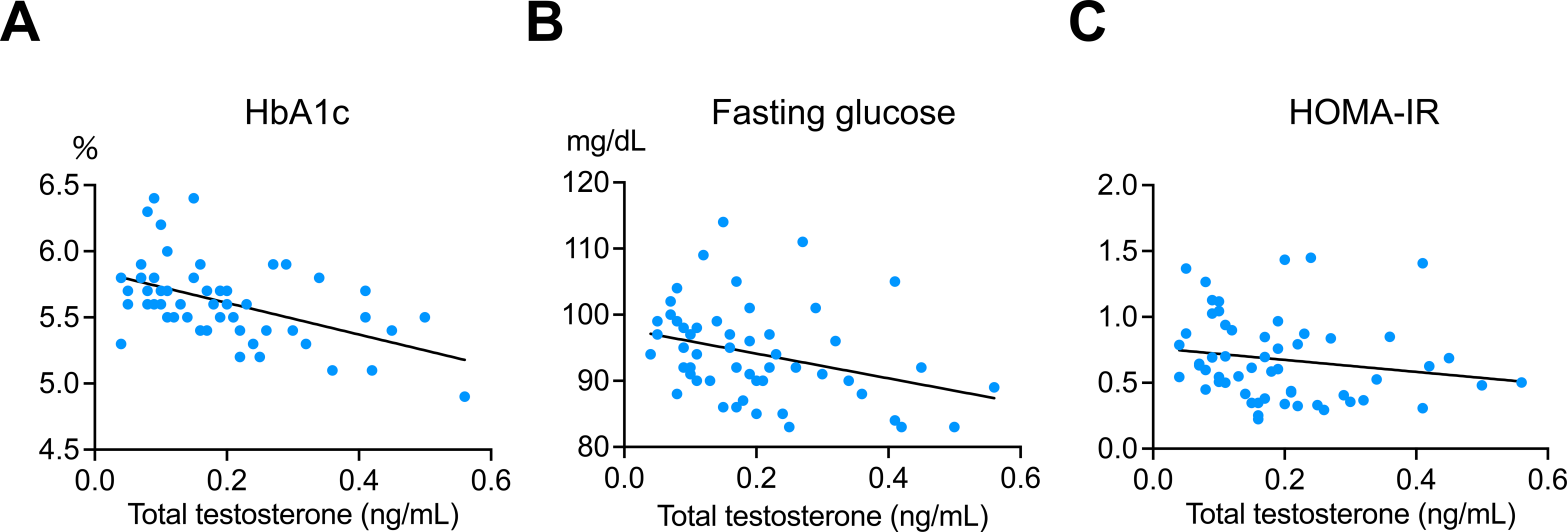
Correlation between serum total testosterone levels and glucose metabolism parameters. The scatter plots demonstrate the associations between serum total testosterone levels and **(A)** HbA1c, **(B)** fasting glucose, and **(C)** HOMA-IR. For clarity of visual representation, only the data from the subjects with values above the limit of detection (LOD) are plotted, and the solid lines represent the linear regression calculated from these detectable values. HbA1c, hemoglobin A1c; HOMA-IR, homeostasis model assessment of insulin resistance.

In normal-weight women, serum total testosterone levels showed significant inverse correlations with HbA1c (*ρ* = -0.341, *p* = 0.004), fasting glucose (*ρ* = -0.327, *p* = 0.006), and HOMA-IR (*ρ* = -0.274, *p* = 0.023) (**Table 2**; **Figures 2**, **3**). These results indicate that higher androgen levels are associated with better systemic insulin sensitivity and glycemic control. Furthermore, serum total testosterone levels were inversely correlated with age (*ρ* = -0.358, *p* = 0.003). In contrast, age showed a positive correlation with glucose metabolism parameters (**Figure 2**).

**Table 2:** Spearman’s rank correlation between serum total testosterone and markers of glucose metabolism.

Parameters	Spearman's *ρ*	*p-value*
HbA1c (%)	-0.341	0.004
Fasting glucose (mg/dL)	-0.327	0.006
HOMA-IR	-0.274	0.023

Correlations between serum testosterone levels and clinical parameters were assessed using Spearman’s rank correlation coefficients.

The statistical analysis included the entire cohort (n = 69), with 13 subjects whose total testosterone levels were below the limit of detection (LOD) being treated as the lowest rank.

HbA1c, hemoglobin A1c; HOMA-IR, homeostasis model assessment of insulin resistance.

Meanwhile, no statistically significant correlations were observed between serum total testosterone levels and other clinical parameters, including systolic/diastolic blood pressure, triglycerides, LDL-C, and HDL-C (all *p* > 0.05; **Supplementary Table 1**).

### 3.3 Multivariate regression analysis of total testosterone and markers of glucose metabolism

Multivariate regression analysis revealed a robust association between serum total testosterone levels and glucose metabolism parameters (**Table 3**). Notably, after adjusting for BMI in Model 1, serum total testosterone was significantly and inversely associated with all parameters: HbA1c (*β* = -0.087, 95% CI: -0.159 to -0.014, *p* = 0.020), fasting glucose (*β* = -1.820, 95% CI: -3.590 to -0.051, *p* = 0.044), and HOMA-IR (*β* = -0.171, 95% CI: -0.320 to -0.023, *p* = 0.024). These significant inverse associations persisted even after further adjustment for lifestyle factors in Model 2. Furthermore, results remained consistent when body fat was used as a covariate instead of BMI; total testosterone showed significant inverse associations with all three glucose metabolism markers after adjustment for lifestyle factors (**Supplementary Table 2**, Models 1 and 2).

**Table 3:** Multivariate regression analysis for the association between serum total testosterone and glucose metabolism markers, adjusted for BMI.

Model	HbA1c (%)		Fasting glucose (mg/dL)		HOMA-IR	
	*β* (95% CI)	*p-value*	*β* (95% CI)	*p-value*	*β* (95% CI)	*p-value*
Model 1	-0.087 (-0.159 to -0.014)	**0.020**	-1.820 (-3.590 to -0.051)	**0.044**	-0.171 (-0.320 to -0.023)	**0.024**
Model 2	-0.083 (-0.160 to -0.006)	**0.035**	-2.130 (-3.955 to -0.305)	**0.023**	-0.184 (-0.340 to -0.027)	**0.022**
Model 3	-0.029 (-0.093 to 0.036)	0.377	-0.959 (-2.736 to 0.818)	0.285	-0.178 (-0.336 to -0.019)	**0.029**
Model 4	-0.028 (-0.093 to 0.037)	0.395	-0.981 (-2.770 to 0.808)	0.278	-0.173 (-0.331 to -0.015)	**0.032**

Values are presented as standardized *β* coefficients with 95% confidence intervals (CIs). Multivariate regression analysis was performed with total testosterone as the independent variable and glycemic markers (HbA1c, fasting glucose, and HOMA-IR) as dependent variables.

Model 1: Adjusted for BMI.

Model 2: Adjusted for BMI and lifestyle factors (smoking status, alcohol intake frequency, and regular exercise habit).

Model 3: Adjusted for BMI and age.

Model 4: Adjusted for BMI, age, and menopausal status.

To account for non-normal distributions, total testosterone, HOMA-IR, BMI, and age were log-transformed prior to analysis.

For the 13 subjects with levels below the LOD, a value of LOD/√2 was imputed for statistical purposes.

Lifestyle factors were assessed and categorized for inclusion in the multivariate models as follows: smoking status was classified into three groups (0, never; 1, former; and 2, current smokers); alcohol intake frequency was scored on a 5-point scale (0, none; 1, 1 day/week; 2, 2–3 days/week; 3, 4–5 days/week; and 4, almost daily); and regular exercise habit was defined as a binary variable (0, no; 1, yes).

BMI, body mass index; HbA1c, hemoglobin A1c; HOMA-IR, homeostasis model assessment of insulin resistance.Bold values indicate statistical significance (*p* < 0.05).

However, when age was included as a covariate in Model 3, the associations with HbA1c and fasting glucose were attenuated and lost statistical significance. In contrast, the inverse association between serum total testosterone and HOMA-IR remained consistently significant (*β* = -0.178, 95% CI: -0.336 to -0.019, *p* = 0.029). Furthermore, in Model 4, which adjusted for menopausal status in addition to the variables included in Model 3, HOMA-IR remained the only marker with a significant independent association (*β* = -0.173, 95% CI: -0.331 to -0.015, *p* = 0.032). These findings emphasize that although age influences overall glycemic markers, serum total testosterone levels are independently and inversely associated with insulin resistance, regardless of BMI or menopausal status.

### 3.4 Comparison of clinical characteristics between premenopausal and postmenopausal states

As a subanalysis to further elucidate the influence of hormonal shifts on metabolic parameters, participants were stratified into premenopausal (n = 31) and postmenopausal (n = 38) groups (**Supplementary Table 3**). No significant differences were observed between the two groups in BMI (21.1 ± 1.8 vs. 21.2 ± 1.7 kg/m², *p* = 0.961) or body fat (29.6 ± 3.7% vs. 28.9 ± 4.4%, *p* = 0.524). Regarding glucose metabolism, HbA1c (5.5 ± 0.2% vs. 5.8 ± 0.3%, p < 0.001) and fasting glucose (92.4 ± 6.6 vs. 96.2 ± 7.6 mg/dL, *p* = 0.033) were significantly higher in the postmenopausal group, along with a trend toward increased HOMA-IR [0.5 (0.4–0.8) vs. 0.7 (0.5–1.0), *p* = 0.069]. Crucially, serum total testosterone levels were significantly lower in the postmenopausal group than in the premenopausal group [0.20 (0.10–0.30) vs. 0.10 (0.05–0.17) ng/mL, *p* = 0.005].

## 4 Discussion

### 4.1 Main results

In this study of normal-weight Japanese women, serum total testosterone levels showed significant inverse correlations with all measured glucose metabolism markers, including HbA1c, fasting glucose, and HOMA-IR. These findings suggest that higher levels of serum total testosterone may exert a protective effect on systemic glucose metabolism in this population. Notably, multivariate regression analysis demonstrated that these associations remained robust even after adjusting for potential confounding factors, including BMI and body fat. The persistence of these relationships regardless of adiposity measures underscores the potential physiological importance of total testosterone in maintaining glycemic control among normal-weight women.

### 4.2 Comparison with findings of previous studies and potential mechanisms

Previous studies have primarily focused on Western, South Asian, and Middle Eastern populations, which are predominantly composed of individuals with obesity or those diagnosed with PCOS (3, 5, 7, 18). These studies demonstrated that excessive androgens induce insulin resistance, which has led to a strong emphasis on the detrimental effects of hyperandrogenism in women. However, our study demonstrates that testosterone deficiency independently correlates with impaired glucose metabolism in normal-weight individuals. This discrepancy suggests the possibility that physiological androgen signaling is protective, whereas its excess exerts detrimental effects.

Several underlying mechanisms may explain these differences. First, testosterone exerts direct protective effects via AR signaling. Studies utilizing tissue specific AR knockout mice revealed that physiological AR activation maintains metabolism in skeletal muscle (12, 19), suppresses gluconeogenesis in the liver (20), and protects insulin secretion in pancreatic β cells (11, 21). However, these mechanisms were predominantly validated in male models, and evidence in females remains insufficient. Second, appropriate levels of testosterone serve as a precursor for local estrogen synthesis via aromatization. This localized estrogen signaling protects pancreatic β cells (22), suppresses hepatic gluconeogenesis, and inhibits visceral fat accumulation (4, 23). Thus, a decline in testosterone may induce a localized deficiency in estrogen action, accelerating metabolic dysfunction.

Conversely, when testosterone levels exceed the physiological threshold, excess testosterone drives insulin resistance through distinct pathways. Studies using female mouse models of androgen excess demonstrate that surplus testosterone induces insulin resistance via complex impacts on the β cells and adipose tissue (24–26).

Taken together, these findings underscore that the physiological action of testosterone and the pathological impact of its excess operate through fundamentally distinct pathways. A clinical parallel exists with thyroid hormone, where physiological levels are essential for metabolic homeostasis, yet its excess drives an entirely different pathological state (13). Similarly, rather than merely representing different degrees of the same condition, maintaining physiological levels of testosterone is a crucial, independent requirement for preserving glucose homeostasis in normal-weight women. Our results align with recent studies including normal-weight participants (27), which suggest that lower levels of total testosterone are associated with an increased risk of metabolic syndrome. This cumulative evidence underscores a paradigm shift from the conventional view of androgens as purely detrimental factors in females, highlighting the clinical significance of maintaining physiological testosterone levels in this population (4, 27).

### 4.3 Interpretations

#### 4.3.1 Research implications

In the present study, serum total testosterone levels exhibited significant correlations with glycemic markers. Although the associations with HbA1c and fasting glucose were attenuated after adjusting for age, suggesting that these parameters may predominantly reflect age-related metabolic shifts, the inverse association with HOMA-IR remained robust and independent of age. This finding implies that maintaining physiological testosterone levels could be a key factor in preventing insulin resistance among normal-weight women experiencing physiological androgen decline, including those who are postmenopausal or have undergone oophorectomy.

Crucially, our subanalysis revealed that postmenopausal women in this cohort had significantly lower serum total testosterone levels alongside worsened glycemic parameters compared with premenopausal women. In heavier populations, literature indicates that testosterone levels decrease gradually with age but are not abruptly altered by menopause (28). However, because our study focused exclusively on normal-weight individuals, their underlying hormonal dynamics appear somewhat distinct. While obese individuals possess abundant adipose tissue that functions as a major site for *de novo* androgen synthesis—thereby buffering local conversion and maintaining circulating levels—normal-weight individuals lack such redundant adipose mass to sustain a self-sufficient turnover (29). Following the postmenopausal decline in ovarian estradiol, peripheral aromatase expression and activity characteristically increase (29, 30). In normal-weight women with limited endogenous androgen-producing capacity, this increased aromatase activity forces an obligatory compensatory shift presumed to actively exhaust the limited circulating androgen pool for estrogen conversion. Consequently, this localized metabolic demand may manifest as the prominent, abrupt reduction in serum total testosterone. Importantly, even after adding both menopausal status and age as adjusting factors in our multivariate regression models, the inverse correlation between testosterone and HOMA-IR remained highly significant. This strongly suggests that maintaining testosterone levels holds a potential role in preventing insulin resistance, rather than being a mere bystander of chronological aging.

The clinical significance of these results is particularly salient for East Asian populations, who are predisposed to glucose intolerance even in the absence of clinical obesity. Given the rapidly increasing elderly female population in East Asia, addressing metabolic health has become an urgent clinical priority (31, 32). In this light, “maintaining physiological testosterone levels” emerges as a critical, yet historically overlooked, strategy for metabolic preservation in aging women. To further substantiate these findings, large-scale observational studies specifically targeting women with physiologically declining testosterone levels, such as postmenopausal and elderly women, are indispensable. Such investigations will help determine whether maintaining androgen levels within the normal physiological range can effectively mitigate the risk of type 2 diabetes and metabolic syndrome in these vulnerable populations.

Furthermore, these studies must account for ethnic variations in androgen sensitivity. Notably, Asian populations tend to harbor longer CAG repeats in the AR gene than other ethnicities (33), which may result in inherently lower AR transactivation activity. This genetic background could explain a potentially lower susceptibility to the adverse effects of androgen excess in Asians. Consequently, broad-scale comparative research across diverse ethnic groups, including Caucasians, African Americans, and Hispanics, is essential to clarify whether the protective effects observed in this study are universal or are significantly modulated by ethnic-specific genetic landscapes and lifestyle factors.

#### 4.3.2 Impact on future research

In existing research in this field, the relationship between androgens and glucose metabolism in normal-weight women has remained a ‘black box’ due to biased study populations. Our results suggest the importance of maintaining physiological androgen levels. Furthermore, these findings offer a new perspective on the concerns regarding metabolic worsening in transgender medicine and androgen replacement therapy for female sexual dysfunction (34, 35). To improve future exploratory studies, several technical and methodological points should be addressed. For instance, many samples fell below the detection limit of the ECLIA method commonly used in male clinical settings, indicating that future studies should utilize LC-MS/MS for higher sensitivity and precision in measuring low-level androgens. Additionally, although this study minimized seasonal variability by unifying sample collection within a single season (winter), it is known that female testosterone levels can exhibit significant seasonal fluctuations (16). Therefore, future research must account for these variations in their sampling protocols to ensure the generalizability of findings across the entire year. Additionally, our subanalysis yielded critical insights into the differences in hormonal and metabolic parameters between premenopausal and postmenopausal women. However, because these stratified evaluations were not explicitly anticipated in the initial study design, the sample sizes for the individual subgroups were not optimized beforehand. To fully validate these important peri-menopausal dynamics with sufficient statistical power, future studies must deliberately incorporate this stratification into their initial sample size calculations and recruit a larger, appropriately balanced cohort.

Ultimately, through this exploratory approach, the present study provides essential foundational data that offer critical insights for future research planning. These findings will inform the strategic design of large-scale, prospective cohort studies, which are necessary to increase statistical power and validate these novel perspectives across broader and more diverse populations.

### 4.4 Strengths and limitations

The study approach itself constitutes a distinct methodological and clinical strength. The primary strength lies in our novel cohort selection strategy that has been largely overlooked in previous literature. Through this design of limiting our study population to normal-weight women, we eliminated the confounding effects and biases associated with obesity. This approach not only allowed us to demonstrate the independent impact of physiological serum total testosterone levels on reduced insulin resistance but also exerts an impact on future research, serving as a methodological reference for the design of future studies on glucose metabolism in women.

However, several limitations must be acknowledged. First, the cross-sectional design precludes any causal inferences regarding the association between testosterone and glucose metabolism. Second, the lack of data concerning menstrual cycles and estradiol levels prevented a complete analysis of the estrogenic pathway, through which androgens might indirectly exert protective effects via aromatization. Third, while elevated sex hormone-binding globulin (SHBG) levels are clinically recognized to be associated with improved glucose metabolism, SHBG itself may contribute to the improvement of insulin resistance, independent of its classical role as a carrier protein. Therefore, the confounding effect of SHBG warrants careful consideration in future analyses. Fourth, the total sample size of this study is relatively small (n = 69), which limits the statistical power of our multivariate and mediation analyses. This limited sample size was insufficient to perform a fully powered, comprehensive stratified analysis based on menopausal status. Therefore, our findings must be interpreted with caution and should be considered preliminary. Future large-scale, multi-center prospective studies with larger cohorts are warranted to further validate the independent protective role of physiological testosterone in female glucose homeostasis. Additionally, this study did not evaluate other androgenic fractions or derivatives, such as bioavailable testosterone, free testosterone, dihydrotestosterone, or dehydroepiandrosterone sulfate (DHEAS). These factors are critical for a more comprehensive understanding of the androgenic milieu and its metabolic consequences.

### 4.5 Perspectives

Despite these limitations, our findings suggest that maintaining total testosterone levels within the physiological range may play a protective role in glucose metabolism among normal-weight women. This exploratory study provides a foundation that may influence the design of large-scale prospective trials aimed at elucidating the potential physiological significance of androgen action in female metabolic health. By highlighting the potential benefits of maintaining physiological androgen levels, this research opens a new frontier in the strategies for maintaining and promoting women’s health, particularly through the understanding and potential management of insulin resistance in non-obese populations.

## Data Availability

The raw data supporting the conclusions of this article will be made available by the authors, without undue reservation.
