# Supplementary material for: Potential role of maintaining physiological testosterone levels in improving glucose metabolism among normal-weight Japanese women: a pilot exploratory study
Source: Front Endocrinol (Lausanne). 2026 Jul 6;17:1878776. doi: 10.3389/fendo.2026.1878776 (PMC13381460; doi:10.3389/fendo.2026.1878776)
Supplement: Supplementary file 1 [file DataSheet1.docx]

Supplementary Material

## Supplementary Tables

**Table S1.** Spearman’s rank correlation between serum total testosterone and markers of glucose metabolism.

| Parameters | Spearman's *ρ* | *p-value* |
| --- | --- | --- |
| Systolic blood pressure (mmHg) | 0.003 | 0.980 |
| Diastolic blood pressure (mmHg) | 0.0004 | 0.997 |
| Triglycerides (mg/dL) | -0.069 | 0.574 |
| Total cholesterol (mg/dL) | -0.227 | 0.061 |
| HDL-cholesterol (mg/dL) | -0.072 | 0.555 |
| LDL-cholesterol (mg/dL) | -0.144 | 0.236 |

Correlations between serum testosterone levels and clinical parameters were assessed using Spearman’s rank correlation coefficients.

The statistical analysis included the entire cohort (n = 69), with 13 subjects whose total testosterone levels were below the limit of detection (LOD) being treated as the lowest rank.

HDL-cholesterol, high-density lipoprotein cholesterol; LDL-cholesterol, low-density lipoprotein cholesterol.

**Table S2.** Multivariate regression analysis for the association between serum total testosterone and glucose metabolism parameters, adjusted for body fat.

| Model | HbA1c (%) | | Fasting glucose (mg/dL) | | HOMA-IR | |
| --- | --- | --- | --- | --- | --- | --- |
|  | *β*  　(95% CI) | *p-value* | *β*  (95% CI) | *p-value* | *β*  (95% CI) | *p-value* |
| Model 1 | -0.086  (-0.160 to -0.013) | **0.022** | -1.801  (-3.602 to -0.0005) | **0.050** | -0.171  (-0.323 to -0.019) | **0.028** |
| Model 2 | -0.084  (-0.162 to -0.006) | **0.036** | -2.186  (-4.063 to -0.309) | **0.023** | -0.191  (-0.352 to -0.029) | **0.021** |
| Model 3 | -0.027  (-0.092 to 0.038) | 0.403 | -0.912  (-2.717 to 0.893) | 0.317 | -0.175  (-0.337 to -0.013) | **0.035** |
| Model 4 | -0.027  (-0.092 to 0.038) | 0.412 | -0.920  (-2.736 to 0.896) | 0.315 | -0.173  (-0.333 to -0.012) | **0.035** |

Values are presented as standardized *β* coefficients with 95% confidence intervals (CIs). Multivariate regression analysis was performed with total testosterone as the independent variable and glycemic markers (HbA1c, fasting glucose, and HOMA-IR) as dependent variables.

Model 1: Adjusted for body fat.

Model 2: Adjusted for body fat and lifestyle factors (smoking status, alcohol intake frequency, and regular exercise habit).

Model 3: Adjusted for body fat and age.

Model 4: Adjusted for body fat, age, and menopausal status.

To account for non-normal distributions, total testosterone, HOMA-IR and age were log-transformed prior to analysis.

For the 13 subjects with levels below the LOD, a value of LOD/√2 was imputed for statistical purposes.

Lifestyle factors were assessed and categorized for inclusion in the multivariate models as follows: smoking status was classified into three groups (0, never; 1, former; and 2, current smokers); alcohol intake frequency was scored on a 5-point scale (0, none; 1, 1 day/week; 2, 2–3 days/week; 3, 4–5 days/week; and 4, almost daily); and regular exercise habit was defined as a binary variable (0, no; 1, yes).

HbA1c, hemoglobin A1c; HOMA-IR, homeostasis model assessment of insulin resistance.

**Table S3**. Clinical characteristics of normal-weight women.

|  | *Premenopausal* (*n*=31) | *Postmenopausal* (*n*=38) | *p-value* |
| --- | --- | --- | --- |
| Age (years) | 45.0 [36.0–48.0] | 63.5 [56.0–68.0] | < 0.001 |
| BMI (kg/m^2^) | 21.1 ± 1.8 | 21.2 ± 1.7 | 0.961 |
| Body fat (%) | 29.6 ± 3.7 | 28.9 ± 4.4 | 0.524 |
| HbA1c (%) | 5.5 ± 0.2 | 5.8 ± 0.3 | < 0.001 |
| Fasting glucose (mg/dL) | 92.4 ± 6.6 | 96.2 ± 7.6 | 0.033 |
| HOMA-IR | 0.5 [0.4–0.8] | 0.7 [0.5–1.0] | 0.069 |
| Total testosterone (ng/mL) | 0.20  [0.10–0.30] | 0.10  [0.05–0.17] | 0.005 |

Values are presented as mean ± SD or median (25th–75th percentiles).

BMI, body mass index; HbA1c, hemoglobin A1c; HOMA-IR, homeostasis model assessment of insulin resistance.
